# The Role of Social Media for Identifying Adverse Drug Events Data in Pharmacovigilance: Protocol for a Scoping Review

**DOI:** 10.2196/47068

**Published:** 2023-08-02

**Authors:** Su Golder, Karen O'Connor, Yunwen Wang, Graciela Gonzalez Hernandez

**Affiliations:** 1 Department of Health Sciences University of York York United Kingdom; 2 Department of Biostatistics, Epidemiology and Informatics Perelman School of Medicine University of Pennsylvania Philadelphia, PA United States; 3 Department of Computational Biomedicine Cedars-Sinai Medical Center West Hollywood, CA United States

**Keywords:** adverse event, pharmacovigilance, social media, real-world data, scoping review, protocol, review method, pharmacology, pharmaceutics, pharmacy, adverse drug event, adverse drug reaction

## Abstract

**Background:**

Adverse drug events (ADEs) are a considerable public health burden resulting in disability, hospitalization, and death. Even those ADEs deemed nonserious can severely impact a patient’s quality of life and adherence to intervention. Monitoring medication safety, however, is challenging. Social media may be a useful adjunct for obtaining real-world data on ADEs. While many studies have been undertaken to detect adverse events on social media, a consensus has not yet been reached as to the value of social media in pharmacovigilance or its role in pharmacovigilance in relation to more traditional data sources.

**Objective:**

The aim of the study is to evaluate and characterize the use of social media in ADE detection and pharmacovigilance as compared to other data sources.

**Methods:**

A scoping review will be undertaken. We will search 11 bibliographical databases as well as Google Scholar, hand-searching, and forward and backward citation searching. Records will be screened in Covidence by 2 independent reviewers at both title and abstract stage as well as full text. Studies will be included if they used any type of social media (such as Twitter or patient forums) to detect any type of adverse event associated with any type of medication and then compared the results from social media to any other data source (such as spontaneous reporting systems or clinical literature). Data will be extracted using a data extraction sheet piloted by the authors. Important data on the types of methods used (such as machine learning), any limitations of the methods used, types of adverse events and drugs searched for and included, availability of data and code, details of the comparison data source, and the results and conclusions will be extracted.

**Results:**

We will present descriptive summary statistics as well as identify any patterns in the types and timing of ADEs detected, including but not limited to the similarities and differences in what is reported, gaps in the evidence, and the methods used to extract ADEs from social media data. We will also summarize how the data from social media compares to conventional data sources. The literature will be organized by the data source for comparison. Where possible, we will analyze the impact of the types of adverse events, the social media platform used, and the methods used.

**Conclusions:**

This scoping review will provide a valuable summary of a large body of research and important information for pharmacovigilance as well as suggest future directions of further research in this area. Through the comparisons with other data sources, we will be able to conclude the added value of social media in monitoring adverse events of medications, in terms of type of adverse events and timing.

**International Registered Report Identifier (IRRID):**

PRR1-10.2196/47068

## Introduction

Adverse drug events (ADEs) cause significant harm by influencing morbidity and mortality and increasing the economic burden on the health care system [1,2]. Furthermore, patients may prematurely discontinue treatment or hesitate to begin new drug treatments due to ADEs, depriving them of the potential beneficial treatment [3]. Not all adverse drug reactions can be detected before a drug is marketed; therefore, continuous surveillance and monitoring of their safety are important.

Traditionally, postmarketing pharmacovigilance for drug safety signals has relied on spontaneous reporting to regulatory agencies. Conventional pharmacovigilance systems have many limitations; most notably, the underreporting of ADEs experienced [4-7]. The low rate of reporting to these agencies has prompted researchers to examine other potential data resources to fill the gap in reporting.

Social media data analysis and monitoring have been investigated for its application in health research including health outcomes research, disease surveillance, and patient perspectives [8-10]. However, the most commonly studied outcome is that of safety [8-10]. Indeed, social media has been identified as a content-rich resource of patient reports of ADEs [11]. Given the volume, velocity, and variety of social media big data across platforms, using social media as a type of supplementary data source may be invaluable to provide perspectives of patients who are otherwise not usually reached through traditional pharmacovigilance channels. The combined use of different data sources may increase the representativeness and comprehensiveness of ADE synthesis by including ADEs experienced and reported by social media users, part of whom can belong to medically underresourced communities that tend to be neglected in regulatory agencies’ reporting programs.

Researchers began exploring the potential value of retrieving drug safety data from social media as early as 2010 [11]. It was believed that social media data could be used to identify new signals or signals earlier than conventional methods [12]. To cope with the enormous amounts of text-based information posted on social media, natural language processing (NLP) and machine learning (ML) methods for automatic detection of mentions are continually being developed [13,14]. These methods have to overcome many challenges, for instance, the language in social media is highly informal, and user-expressed concepts are often nontechnical, descriptive, and challenging to extract [15,16]. NLP has been particularly instrumental in overcoming some of the barriers to identify adverse event mentions [13,14]. However, while the technological methods have advanced, the use of social media in identifying adverse events has not been sufficiently demonstrated, and thus, the debate as to whether (and if so, how) social media can enhance pharmacovigilance is still not resolved.

While many papers have concluded that social media has the potential to improve pharmacovigilance, others have argued that this is not the case, including the well-known Web-Recognizing Adverse Drug Reactions study [17]. These papers have tended to rely on case studies and the selection of these case studies, and the comparative analysis used may have impacted the results. The question that needs answering may be more complex and nuanced than whether or not social media can be used in pharmacovigilance, necessitating not a binary yes or no answer. The question may rather be how and in what circumstances social media can improve pharmacovigilance and when social media is less appropriate or even not appropriate in pharmacovigilance.

In total, 7 systematic reviews have been published from 2015 to 2021 that attempted to evaluate the potential use of social media in pharmacovigilance by either focusing on detection, such as safety signals, or the frequency of adverse event reports [12,18-23] (Table 1). These reviews, despite including over a hundred papers, largely concluded that the research is still in its infancy and that more research is required. Some of the reviews, however, did highlight that social media was more appropriate for mild symptomatic ADEs and for gaining patient perspectives of salient events and their impact or identifying adverse event signals earlier than regulatory agencies. The methods used to extract data from social media are continuously improving, and many more studies have been published since these reviews were published. These reviews also focused on ADE detection for pharmacovigilance or extraction methods, but they did not compare the types of drugs or the specific adverse events for which social media may prove more successful.

**Table 1:** Systematic or scoping reviews of the use of social media for pharmacovigilance.

Author (year)	Date searched	Included studies, n	Studies with comparison	Main focus of included studies	Review conclusions
Golder et al (2015) [18]	2015	51	29	ADE^a^ detection	Higher frequency of adverse events was found in social media and that this was particularly true for symptom-related and mild adverse events. Reliability or validity not thoroughly evaluated.
Lardon et al (2015) [23]	2014	24	6	ADE detection (11 papers) and extraction methods (13 papers)	Detection: studies failed to accurately assess the completeness, quality, and reliability of the data. Extraction: no study proposed a generic approach to easily adding a new site or data source. Additional studies are required.
Sarker et al (2015) [19]	2015	22	10	Extraction methods	Annotated data are publicly available in a still very limited amount. As indicated by the promising results obtained by recent supervised learning approaches, there is a strong need to make such data available to the research community.
Tricco et al (2018) [12]	May 2016	70	19	ADE detection	Social media is potential to supplement data from regulatory agency databases; it can capture less frequently reported AEsb and identify AEs earlier than official alerts or regulatory changes, but the use and validity of the data source remain understudied.
Convertino et al (2018) [20]	December 2017	38	38	ADE signal detection	Poorer information quality as compared with spontaneous reporting databases. Rarely allows the evaluation of causal relationships. Proto signals had the potential of anticipating prespecified known signals in only 6 studies. Social media is not currently recommended for pharmacovigilance.
Pappa and Stergioulas (2019) [21]	Early 2018	100	Not reported	AE signal detection	Developed new classifications of social data sources and taxonomies for social data, identified key challenges, and extracted new insights in terms of potential for practical applications.
Lee et al (2021) [22]	January 2021	14	14	ADE signal detection	Assessed the time interval between signals detected on social media and regulatory authority action, revealing that most studies reported that meaningful signals could be identified 3 months to 9 years before regulatory authorities take action.

^a^ADE: adverse drug event.

^b^AE: adverse event.

We propose to undertake an updated analysis that allows us to validate the necessary conditions and methods to harness adverse event reports from social media as a complement to other sources. Given the wide breadth of studies conducted in this area and our objective to summarize the literature, we propose to undertake a scoping review using the Arksey and O’Malley [24] framework to answer the following questions: (1) what recent research has been undertaken into the detection of adverse events from social media at a large scale? (2) What types of drugs and adverse events have been studied to date with what findings? (3) How do the types and frequency of ADEs identified from social media differ from those identified from other sources, such as regulatory data or clinical trials? (4) What extraction methods are used to identify ADE data from social media and what impact may this have on the results?

## Methods

### Study Design

This scoping review protocol is reported in line with the PRISMA-ScR (Preferred Reporting Items for Systematic Reviews and Meta-Analyses Extension for Scoping Reviews) checklist [25]. The inclusion and exclusion criteria present in Textbox 1 will be used in order to provide an understanding of the volume of the research in this area.

**Inclusion and exclusion criteria for studies on identifying adverse drug events data from social media.**

**Inclusion criteria**

- Population
  Any person (including pregnant women and young and older adults) with or without any condition or disease type (chronic or acute) states that they have taken any drug intervention (including vaccines) used in diagnosis, treatment, or prevention (as defined by the Food and Drug Administration [FDA]) and experienced an adverse event.
- Interventions
  Any type of social media defined as any computer-mediated tools for users to create, share, or exchange information, ideas, or content via text, images, and audio (eg, message postings, pictures, and videos) in web-based communities and networks (such as message boards, social networks, patient forums, Twitter, Reddit, blogs, and Facebook).
- Comparators
  Any data source other than social media (such as spontaneous reporting systems of the FDA or the Medicines and Healthcare products Regulatory Agency, clinical trials, or summary of product characteristics) is eligible as a comparator.
- Outcomes
  Primary outcomes: Data on the type and frequency of adverse drug events (such as muscle aches, headaches, or rashes) are required from social media and at least one other data source.
  Secondary outcomes: Data on the application of the adverse drug events (such as pharmacovigilance and hypothesis generation).
- Study design
  Any type of assessment.
- Limits
  Published 2017 onward in English, Spanish, French, or Chinese or in any language with an English translation available.

**Exclusion criteria**

- Population
  Reports by health care professionals.
  People reporting diagnosis, treatment, or prevention with a nonmedical intervention (such as medical devise, surgery, supplements, or natural remedy). People not reporting experience of an adverse event.
- Interventions
  Simple, nonsocial internet-based interventions (ie, web 1.0).
  Studies using social media to recruit participants.
- Comparators
  No comparison undertaken to any nonsocial media data source.
- Outcomes
  We are concerned with the properties of interventions under normal use. We therefore will not consider papers where the primary aim was to assess events such as intentional and accidental poisoning (ie, overdose), drug abuse, errors, or noncompliance. Drug-drug interactions are not eligible where they are the primary objective of the paper due to the different techniques required in identifying interactions as opposed to adverse events under normal use.
  Papers focused on identifying patient perspectives of adverse events (such as fear or impact on quality of life) and papers on subsequent patient behaviors as a result of adverse events are also ineligible.
- Study design
  Discussion papers, purely technical papers, and papers that only contain examples of posts from social media.
- Limits
  Anything published before 2017 and anything published since 2017 that is not in English, Spanish, French, or Chinese or in another language with no available English translation.

### Search Methods

In total, 11 databases covering a range of topic areas, including health and medical sciences, nursing, information and computer science, and gray literature will be searched. We will also search Google Scholar; however, due to the immense number of hits this will retrieve, we will only sift the first 300 records [26]. Searching databases may not retrieve all relevant available studies as there are delays in indexing, they may not have been indexed adequately (particularly where the database does not index using full text or uses automated methods) or they may have a lack of detail in titles and abstracts. We will, therefore, conduct handsearching of the most common journal titles from a previous review [18]: Drug Safety; Journal of Medical Internet Research, and Pharmacoepidemiology and Drug Safety (Textbox 2).

**Sources to be searched.**

**Databases**

- ACM Digital Library
- Conference Proceedings Citation Index—Science
- Emerging Sources Citation Index
- Embase
- IEEE Xplore
- Library, Information Science & Technology Abstracts
- MEDLINE
- OpenDissertations
- ProQuest Dissertations & Theses: United Kingdom and Ireland
- PsycINFO
- Science Citation Index Expanded

**Internet search engine**

- Google Scholar (first 300 records will be sifted)

**Handsearching of journals**

- *Drug Safety* (2017-2023)
- *Journal of Medical Internet Research* (2017-2023)
- *Pharmacoepidemiology and Drug Safety* (2017-2023)

The database search strategies will have 2 facets—“social media” and “adverse events” (see Multimedia Appendix 1 for draft search strategy in Ovid MEDLINE, which will be translated for other databases and interfaces as necessary).

A publication date restriction of 2017 onward will be placed on the searches as this review updates 7 previous reviews [12,18-23], the most recent of which is more focused than our review [22]. No language restrictions will be placed on the searches, although financial and logistical restraints will not allow translation from all languages; therefore, this review will be likely to focus more on papers published in English, Spanish, French, or Chinese and other languages for which an English language translation is available. We will also undertake forward and backward citation searching by checking the references of all included studies and forward citation searching using CitationSearcher to identify papers that have cited key papers we identify. Searches will be rerun before conducting the final analysis to retrieve any further includable studies.

The results of all the searches will be entered into an EndNote (Clarivate) library with the duplicates removed. Title and abstract screening will be undertaken independently by 2 reviewers in Covidence (Covidence) with any disagreements resolved by discussion or if necessary, a third reviewer. Full-text screening will again be undertaken in Covidence by 2 independent reviewers.

### Data Extraction

A data extraction spreadsheet will be designed and piloted for this review. The form will record study characteristics of existing papers on using social media data to identify potential ADEs. Two reviewers will extract descriptive data independently, with findings compared and agreed. The following data will be extracted from included studies if available: (1) details on the type of social media platform used; (2) details on the primary aim of the study; (3) brief details of the methods used to extract data from social media including which drugs or adverse events are searched for and how and for what time period; (4) whether the study distinguished between personal and nonpersonal mentions, and whether it accounted for the influence of bots or nonindividual accounts; (5) the type and frequency of adverse events data identified for each drug and which drug; (6) comparator data sources along with any comparisons of the data collected; (7) conclusions of the original investigators; and (8) finally, whether code or annotated or raw data are made available by the authors.

As this is a scoping review, we will not assess the methodological quality (risk of bias assessment) of the studies or conduct any evidence synthesis. Nevertheless, we will summarize an array of NLP and ML (artificial intelligence) methods used for this task and synthesize the studies’ self-reported performances and if available, scalability per method.

### Ethical Considerations

Since the scoping review methodology consists of reviewing and collecting data from publicly accessible materials, this study does not require ethics approval.

## Results

Unlike a systematic review, a scoping review does not “synthesize” the evidence or aggregate results from the included studies [24]. Although this scoping review does not involve raw data synthesis summary across all studies to be reviewed, descriptive statistics with each study as a unit of analysis will be used. Tables, graphs, and charts will be provided when applicable regarding the range of social media platforms evaluated, the frequency and types of adverse events and drugs identified, the types of methods used (including data collection), the types of data comparisons made, the data source compared to, as well as the results and conclusions. We will also require some form of analytic framework. The literature will be organized by type of comparison made (eg, spontaneous reporting systems, clinical literature, and summary of product characteristics). We will also seek to summarize our analysis by type of social media, type of adverse events, and type of ADE detection or extraction methods if applicable.

We will compare our results of studies from 2017 onward to the results of the 7 systematic reviews identified. Any discrepancies between the results will be investigated in terms of methods used and any other possible explanations.

## Discussion

### Principal Findings

Through the use of ML and NLP techniques, the automatic detection and extraction of ADEs from social media have been the focus of much research to refine the methods, including shared task competitions in the community [27]. This review will update and build upon previous reviews in this rapidly evolving field. Especially, the contributions to the current literature will be surrounding 2 contextual questions we asked in the beginning, namely, how and in what circumstances social media can improve pharmacovigilance and when social media may be less appropriate or even not appropriate in pharmacovigilance. Therefore, part of this review’s objective is to suggest to fellow researchers the optimal use of social media data for ADE detection. Without suggesting that social media is a better or worse approach to pharmacovigilance than alternative data sources, we set this review to assist future research in this area in improving study design, validity, scalability, and reproducibility. We will also provide evidence-based discussions on the conceptual challenges we have identified through this review, the possible solutions to them, and future research directions.

We will review the latest research allowing us to elucidate how recent advances in the fields of ML and NLP are being used to advance discoveries in this research area. We will also address any patterns in the findings related to the methods and drugs and adverse events studied. Additionally, the results will objectively inform the debate regarding the value of social media in identifying adverse events and bring us nearer to a consensus. By systematically evaluating the comparisons made to other sources, we will be able to identify the similarities and differences in what is reported in social media, and by extension, identify the burden of ADEs from the patient’s perspective. We will also highlight any gaps in the evidence and priorities for future research.

We will also discuss any issues that may have risen in terms of access to data subsequent to the time at which the studies were conducted. This will be particularly relevant given the new restrictions some social media platforms have placed on their data or application programming interface availability. For example, whether the data from the selected social media platforms are still available to access, and whether it is free to access or based on a commercial license. We will also discuss our results in the context of user privacy concerns, commercial interests, and barriers to social media research. This review is expected to be of particular use to regulatory agencies, and researchers wanting to prioritize research on suspected adverse events or monitor adverse events.

### Strengths and Limitations

There are 2 main strengths to this study. First, different experts in the fields of NLP, ML, systematic review methodology, and information science will participate in the planning and development of the study. Second, we have already identified 7 previous systematic reviews or scoping reviews on which we can build our methods. The main limitations of our study are the exclusion of studies published in languages other than English, French, Spanish, or Chinese and the use of Anglo-dominated databases. This is also a fast-paced area of research meaning that the applicability of our findings may change over time.

### Conclusions

This scoping review protocol outlines steps that will guide our scoping review. This review will also identify and map studies that indicate the value of social media in detecting adverse events and improving pharmacovigilance. The results of this study may help inform current recommended practices and the future direction of research in this area.

## Appendix

Multimedia Appendix 1 Draft search strategy for Ovid MEDLINE.

## Abbreviations

ADE: adverse drug event
ML: machine learning
NLP: natural language processing
PRISMA-ScR: Preferred Reporting Items for Systematic Reviews and Meta-Analyses Extension for Scoping Reviews

## Data Availability

The full search strategies for all the databases, the data extraction sheets, and a full list of the included and excluded studies will be made available in the completed scoping review.
