# Supplementary material for: The Role of Social Media for Identifying Adverse Drug Events Data in Pharmacovigilance: Protocol for a Scoping Review
Source: JMIR Res Protoc. 2023 Aug 2;12:e47068. doi: 10.2196/47068 (PMC10433020; doi:10.2196/47068)
Supplement: Multimedia Appendix 1 [file resprot_v12i1e47068_app1.docx]

# Multimedia Appendix 1

Draft search strategy for Ovid MEDLINE(R) ALL, to be converted for other databases and interfaces as appropriate:

1 social media.ti,ot,ab,kw,kf.

2 social medias.ti,ot,ab,kw,kf.

3 web 2*.ti,ot,ab,kw,kf.

4 blog*.ti,ot,ab,kw,kf.

5 wiki.ti,ot,ab,kw,kf.

6 wikis.ti,ot,ab,kw,kf.

7 weblog*.ti,ot,ab,kw,kf.

8 web log*.ti,ot,ab,kw,kf.

9 facebook.ti,ot,ab,kw,kf.

10 face book.ti,ot,ab,kw,kf.

11 tweet.ti,ot,ab,kw,kf.

12 tweets.ti,ot,ab,kw,kf.

13 tweeting.ti,ot,ab,kw,kf.

14 twitter*.ti,ot,ab,kw,kf.

15 you tube.ti,ot,ab,kw,kf.

16 youtube.ti,ot,ab,kw,kf.

17 social web.ti,ot,ab,kw,kf.

18 social software.ti,ot,ab,kw,kf.

19 social medium.ti,ot,ab,kw,kf.

20 crowd sourcing.ti,ot,ab,kw,kf.

21 crowdsourcing.ti,ot,ab,kw,kf.

22 instant messaging.ti,ot,ab,kw,kf.

23 microblogging.ti,ot,ab,kw,kf.

24 social bookmark*.ti,ot,ab,kw,kf.

25 patientslikeme.ti,ot,ab,kw,kf.

26 patient forum*.ti,ot,ab,kw,kf.

27 discussion forum*.ti,ot,ab,kw,kf.

28 online forum*.ti,ot,ab,kw,kf.

29 chat forum*.ti,ot,ab,kw,kf.

30 chatforum*.ti,ot,ab,kw,kf.

31 chatroom*.ti,ot,ab,kw,kf.

32 chat room*.ti,ot,ab,kw,kf.

33 (online adj2 discussion*).ti,ot,ab,kw,kf.

34 discussion board*.ti,ot,ab,kw,kf.

35 online chat*.ti,ot,ab,kw,kf.

36 (online adj2 communication*).ti,ot,ab,kw,kf.

37 digital media.ti,ot,ab,kw,kf.

38 digital medias.ti,ot,ab,kw,kf.

39 personal health message*.ti,ot,ab,kw,kf.

40 user comment*.ti,ot,ab,kw,kf.

41 patients posts.ti,ot,ab,kw,kf.

42 users posts.ti,ot,ab,kw,kf.

43 internet accounts.ti,ot,ab,kw,kf.

44 internet sites.ti,ot,ab,kw,kf.

45 message board*.ti,ot,ab,kw,kf.

46 web scale.ti,ot,ab,kw,kf.

47 google plus.ti,ot,ab,kw,kf.

48 user generated.ti,ot,ab,kw,kf.

49 consumer generated.ti,ot,ab,kw,kf.

50 online health content.ti,ot,ab,kw,kf.

51 internet narrative*.ti,ot,ab,kw,kf.

52 social network* site*.ti,ot,ab,kw,kf.

53 online social network*.ti,ot,ab,kw,kf.

54 social networking.ti,ot,ab,kw,kf.

55 online comment*.ti,ot,ab,kw,kf.

56 internet forum*.ti,ot,ab,kw,kf.

57 web forum*.ti,ot,ab,kw,kf.

58 internet media.ti,ot,ab,kw,kf.

59 web media.ti,ot,ab,kw,kf.

60 sentiment analysis.ti,ot,ab,kw,kf.

61 Social Media/

62 Blogging/

63 Crowdsourcing/ 1188

64 health forum*.ti,ot,ab,kw,kf.

65 instagram.ti,ot,ab,kw,kf.

66 linkedin.ti,ot,ab,kw,kf.

67 reddit.ti,ot,ab,kw,kf.

68 mastodon.ti,ot,ab,kw,kf.

69 tiktok.ti,ot,ab,kw,kf.

70 snapchat.ti,ot,ab,kw,kf.

71 spotify.ti,ot,ab,kw,kf.

72 pinterest.ti,ot,ab,kw,kf.

73 telegram.ti,ot,ab,kw,kf.

74 tumblr.ti,ot,ab,kw,kf.

75 wechat.ti,ot,ab,kw,kf.

76 whatsapp.ti,ot,ab,kw,kf.

77 webmd.ti,ot,ab,kw,kf.

78 dailystrength.ti,ot,ab,kw,kf.

79 askapatient.ti,ot,ab,kw,kf.

80 or/1-79 53918

81 (adverse adj2 (interaction$ or response$ or effect$ or event$ or reaction$ or outcome$)).ti,ot,ab,kw,kf.

82 side effect$.ti,ot,ab,kw,kf.

83 (unintended adj2 (interaction$ or response$ or effect$ or event$ or reaction$ or outcome$)).ti,ot,ab,kw,kf.

84 (unintentional adj2 (interaction$ or response$ or effect$ or event$ or reaction$ or outcome$)).ti,ot,ab,kw,kf.

85 (unwanted adj2 (interaction$ or response$ or effect$ or event$ or reaction$ or outcome$)).ti,ot,ab,kw,kf.

86 (unexpected adj2 (interaction$ or response$ or effect$ or event$ or reaction$ or outcome$)).ti,ot,ab,kw,kf.

87 (undesirable adj2 (interaction$ or response$ or effect$ or event$ or reaction$ or outcome$)).ti,ot,ab,kw,kf.

88 (serious adj2 (interaction$ or response$ or effect$ or event$ or reaction$ or outcome$)).ti,ot,ab,kw,kf.

89 (toxic adj2 (interaction$ or response$ or effect$ or event$ or reaction$ or outcome$)).ti,ot,ab,kw,kf.

90 (adrs or ades).ti,ot,ab,kw,kf.

91 drug safety.ti,ot,ab,kw,kf.

92 (drug surveillance or ((postmarketing or post marketing) adj2 surveillance)).ti,ot,ab,kw,kf.

93 product surveillance.ti,ot,ab,kw,kf.

94 drug monitoring.ti,ot,ab,kw,kf.

95 tolerability.ti,ot,ab,kw,kf.

96 treatment emergent.ti,ot,ab,kw,kf.

97 toxicity.ti,ot,ab,kw,kf.

98 pharmacovigilance.ti,ot,ab,kw,kf.

99 drug withdrawal*.ti,ot,ab,kw,kf.

100 ae.fs.

101 to.fs.

102 Product Surveillance, Postmarketing/

103 Adverse Drug Reaction Reporting Systems/

104 pharmacovigilance/

105 Drug Monitoring/

106 exp Drug Hypersensitivity/

107 exp "Drug-Related Side Effects and Adverse Reactions"/

108 Abnormalities, Drug-Induced/

109 Safety-Based Drug Withdrawals/

110 Drug Recalls/

111 safety signal*.ti,ot,ab,kw,kf.

112 or/81-111

113 80 and 112

114 limit 113 to yr="2017 -Current"
